# Interleukin-1α and Interleukin-1β play a central role in the pathogenesis of fulminant hepatic failure in mice

**DOI:** 10.1371/journal.pone.0184084

**Published:** 2017-09-27

**Authors:** Maya Sultan, Ziv Ben-Ari, Rula Masoud, Orit Pappo, Dror Harats, Yehuda Kamari, Michal Safran

**Affiliations:** 1 The Liver Research Laboratory Chaim Sheba Medical Center, Tel Hashomer, Ramat Gan, Israel; 2 The Liver Disease Center Chaim Sheba Medical Center, Tel Hashomer, Ramat Gan, Israel; 3 The Sackler School of Medicine, Tel Aviv University, Tel Aviv, Israel; 4 Department of Pathology Chaim Sheba Medical Center, Tel Hashomer, Ramat Gan, Israel; 5 The Bert W. Strassburger Lipid Center, Chaim Sheba Medical Center, Tel Hashomer, Ramat Gan, Israel; University of Navarra School of Medicine and Center for Applied Medical Research (CIMA), SPAIN

## Abstract

**Background and aims:**

Fulminant hepatitis failure (FHF) is marked by the sudden loss of hepatic function, with a severe life-threatening course in persons with no prior history of liver disease. Interleukin (IL)-1α and IL-1β are key inflammatory cytokines but little is known about their role in the development of FHF. The aim of this study was to assess the involvement of IL-1α and IL-1β in the progression of LPS/GalN-induced FHF.

**Methods:**

WT, IL-1α or IL-1β deficient mice were injected with LPS/GalN. Blood and liver tissue were collected at different time points, FHF related pathways were examined.

**Results:**

After FHF induction the survival of both IL-1α and IL-1β KO mice was longer than that of WT mice. Lower serum liver enzyme levels, demonstrated reduced hepatic injury in the IL-1α and IL-1βKO mice. Histologically detected liver injury and apoptotic hepatocytes were significantly reduced in the IL-1αand IL-1βKO mice compared to WT mice. Reduced hepatic IkB levels and upregulated NFκB activity in WT mice remained inhibited in IL-1α and IL-1β KO mice. Hepatic expression levels of TNFα and IL-6 were significantly increased in WT mice but not in IL-1α and IL-1β KO mice.

**Conclusions:**

IL-1α and IL-1β play a central role in the pathogenesis of LPS/GalN-induced FHF. These interleukins are associated with the activation of NFκB signaling, upregulation of the pro-inflammatory cytokines and liver damage and apoptosis. Since neither IL-1α nor IL-1β depletions completely rescued the phenotype, we believe that IL-1α and IL-1β have a similar and probably complementary role in FHF progression.

## Introduction

Fulminant hepatic failure (FHF) can be a fatal complication of acute hepatic injury and occurs unpredictably. It is a rare clinical entity marked by the sudden onset of hepatic encephalopathy and loss of hepatic function manifested by coagulopathy, jaundice and multisystem organ failure in persons with no prior history of liver disease [1, 2]. The causes of FHF include viral hepatitis, drug-induced and toxin-induced liver damage, metabolic errors, ischemia, and miscellaneous rare causes. FHF has a severe life-threatening course, although a major improvement in survival has been reported due to the implementation of complex intensive care unit protocols and emergency liver transplantation [3]. Sepsis and endotoxemia are common complications, and microbiologically proven infection occurs in up to 80% of FHF patients [4].

FHF is typically induced in animals for research purposes, by administration of D-galactosamine (GalN), together with endotoxin, a gram-negative bacterial lipopolysaccharide (LPS) that releases a wide variety of inflammatory mediators, which are considered to be related to the development of FHF as well as to multiple organ failure [5–7].

The IL1 family of ligands and receptors is primarily associated, more than any other family of cytokines, with acute and chronic inflammation [8]. The main function of IL1–type cytokines is to control pro-inflammatory reactions in response to pathogen-induced tissue injury [9]. Cells of the innate immune system constitute the major source of secreted IL-1α and IL-1β [10, 11]. Other cell types, such as epithelial cells [12], endothelial cells [13], and fibroblasts [14], are also known to produce IL-1α and IL-1β. Macrophages are induced to synthesize IL-1α by pathways that include danger-associated molecular patterns (DAMPs), such as modified lipids and foreign particles, and pathogen-associated molecular patterns (PAMPs), such as LPS [8, 15, 16]. IL-1α is mainly active as an intracellular precursor or in its membrane-associated form; only a small proportion of this protein is active in its secreted form [17]. Recently, it has been shown that following an apoptotic stimuli, the IL-1α precursor translocates to the nucleus where it binds to chromatin and is not released from cells [18]. However, when released from necrotic cells, IL-1α serves as a "danger signal" and induces sterile inflammation manifested by recruitment of myeloid lineage inflammatory cells [19–21]. Although over-expression of IL-1α was reported to induce apoptosis [22], the relevance of these results to apoptosis in vivo remains unclear. We recently reported that IL-1α-deficient mice are protected from atherogenic diet-induced steatohepatitis and from ER stress-induced apoptosis and liver damage [23]. IL-1β is generated as an inactive precursor that is transferred into specialized secretory lysosomes, where it is processed into an active mature form by Caspase 1. The mature IL-1β is released into the extracellular environment [8]. IL1 signal transduction begins upon ligand binding to the receptor (IL1R) and recruitment of the adaptor proteins MyD88 and IRAK4 to the TIR domain [24]. Eventually, this signal transduction pathway leads to upregulation of genes involved in enhanced immune reactivity and/or inflammation [25].

Upon stimulation by LPS, macrophages secrete pro-inflammatory cytokines, including IL-1, IL6, IL12, and TNFα [26]. The production of TNFα and IL-1 was shown to be elevated in FHF patients compared to normal control volunteers. The most significant elevation in IL-1 levels was detected in FHF patients that eventually died from this syndrome [27]. A later study demonstrated high serum IL-1β levels and a significantly reduced ratio between the IL-1 receptor antagonist (IL-1Ra) and IL-1β (IL-1Ra/IL-1β) in patients who died as a result of FHF, when compared to those who survived the syndrome [28]. Introduction of Ad-IL-1Ra (adenovirus IL-1 receptor antagonist), either by direct injection or by way of a bio-artificial liver (BAL) device, to the livers of mice before the induction of FHF, led to a significant reduction in plasma levels of hepatic enzymes and improved animal survival [29]. Yet, little is known about the molecular mechanism of FHF development and the role of IL-1α or IL-1β in the process. This study aimed to characterize the involvement of IL-1α and IL-1β in the pathogenesis of LPS/GalN-induced FHF using wild type (WT) versus IL1α- or IL1β-deficient mice.

## Materials and methods

### Animals and experiment protocols

Wild type (WT) C57BL/6 male mice (Harlan, Israel) and IL-1α knockout (KO) and IL-1β KO mice [30, 31] were maintained in a pathogen-free facility and fed pellet food and water *ad libitum*, until the start of the experiment (at 12 weeks old). All experiments were carried out in accordance with the institutional guidelines for animal care. The experimental protocol was approved by the local ethics committee.

### LPS/GalN-induced FHF

To induce FHF, 10 mg/kg Escherichia coli LPS (a phenol extract of serotype 011:B4) (Sigma St. Louis, MO, USA) and 300 mg/kg GalN (Sigma St. Louis, MO) were intra-peritoneally (IP) administered to mice. Saline was IP injected into control mice.

### Ethic statement

All experiments were carried out in accordance with the institutional guidelines for animal care. The experimental protocol was approved by the Chaim Sheba Medical Center at Tel Hashomer ethics committee.

For the survival study time of death is the most critical endpoint. In this study euthanizing mice prematurely might false the results. Furthermore, since the progression of FHF after the injection of LPS/GalN is very fast and most of the mice died within 6–7 hours we could not use other parameters that will reflect the differences between the different groups of mice, as a humane endpoint. Nevertheless, minimizing animal distress or suffering is of prime importance. Therefore, a balance between minimizing animal pain and avoiding bias in survival curves should be found. Animals’ wellbeing was followed every 30 minutes for signs of distress and endpoints. The overall health status was checked by trained professionals. Specific criteria used to determine when the animals should be euthanized were based on personal experience. Mice were euthanized when they were found in a moribund state as identified by inability to maintain upright associated or not with labored breathing and cyanosis. Euthanized mice were considered as non-survivors.

In all other experiments that mice were scarified within 5 hours so they did not reached "death as an endpoint". All experiments were carried out in accordance with the institutional guidelines for animal care.

### Survival experiment

WT and IL-1α KO or IL-1β KO mice treated with either saline (n = 5 per group) or LPS/GlaN (n = 8 per group) and were monitored every 30 min basis for 24 hours.

### Time-course experiment

Mice receiving saline (n = 1 for each time point) or LPS/GlaN treatment (n = 3 for each time point) were euthanized at 0.5, 1, 1.5, 3, 4 and 5 hours after treatment. Blood samples were collected from the vena cava for serum separation, and liver specimens were either fixated in formalin for histological staining or snap-frozen in liquid nitrogen and stored at -80°C for protein and RNA extraction. Serum aspartate transaminase (AST) and alanine transaminase (ALT) levels were determined using standard procedures. Liver specimens were embedded in paraffin and stained with hematoxylin-eosin (H&E). Pathological findings were assessed by a pathologist blinded to the treatment. ALT and AST levels were measured using BECKMAN COULTER test according to the manufacture instruction"

### TUNEL assay

Apoptotic cells were identified using the DeadEnd^™^ flourometric TUNEL system (Promega Madison, WI USA), applied as per the manufacturer's instructions, and followed by nuclear staining with DAPI (Life Technologies Carlsbad, CA USA).

### Western blot analysis

Liver tissue samples were homogenized in RIPA lysis buffer (Sigma St. Louis, MO USA) containing complete, mini protease inhibitor cocktail tablets (Roche) and phosphatase inhibitors (cocktail2&3) (Sigma St. Louis, MO USA). Protein levels were quantified using a commercial BCA kit (Pierce Waltham, MA USA). Liver protein extracts (40μg protein/ lane) were separated on polyacrylamide gels by SDS-PAGE under reducing conditions, and transferred to nitrocellulose membranes. The membranes were probed with anti-rabbit caspase3 monoclonal antibody (1:1000) (Cell Signaling Danvers, MA USA), anti-rabbit IκB-α polyclonal antibody (1:500) or anti-mouse HSC70 monoclonal antibody (1:10,000) (Santa Cruz Dallas, TX, USA), followed by incubation with HRP-conjugated secondary antibody (1:10,000) (Jackson ImmunoResearch,) and ECL chemiluminescent substrate (Pierce Waltham, MA, USA).

### RNA purification and real-time qPCR analysis

Total RNA was extracted from liver samples using TRI-Reagent (Sigma St. Louis, MO USA), followed by treatment with 1U of RNase-free DNase (Roche Basel, Switzerland). Reverse transcription was performed on 2 μg total RNA using the High Capacity cDNA Reverse Transcription Kit (Applied Biosystems Foster City, CA USA), according to the manufacturer’s instructions. qPCR was performed on 50ng cDNA samples using the SYBR Green Real-Time PCR Kit (Applied Biosystems Foster City, CA USA), according to manufacturer’s specifications, with gene-specific primers (Table 1) in an ABI Step ONE Plus system (Applied Biosystems Foster City, CA USA). HPRT and β-Actin were used as an endogenous reference control. All reactions were performed in duplicates and relative gene expression values were determined using the 2.ddCt method with ABI Prism 7000 SDS (Applied Biosystems Foster City, CA USA). Results from the WT saline group were used as a reference group for the calculations.

**Table 1:** Primers list.

Gene	Forward	Reverse
**m HPRT**	AGCTACTGTAATGATCAGTCAACG	AGAGGTCCTTTTCACCAGCA
**m β Actin**	CCTGTATGCCTCTGGTCGTA	CCATCTCCTGCTCGAAGTCT
**mIL-1α**	GAGTCGGCAAAGAAATCAAGATG	CAATGGCAGAACTGTAGTCTTCGT
**m IL-1β**	CTGCAGCTGGAGAGTGTGGAT	CTCCACTTTGCTCTTGACTTCTATCTT
**m IL-6**	CACATGTTCTCTGGGAAATCG	TTGTATCTCTGGAAGTTTCAGATTGTT
**mTNFα**	GCCACCACGCTCTTCTGTCTAC	GGGTCTGGGCCATAGAACTGAT

### Statistical analysis

Results are expressed as mean ± standard error. Differences between groups were assessed using the analysis of variance (T-TEST).

## Results

### The effect of IL-1α and IL-1β deficiency on mice survival following FHF induction

**Fig 1:**
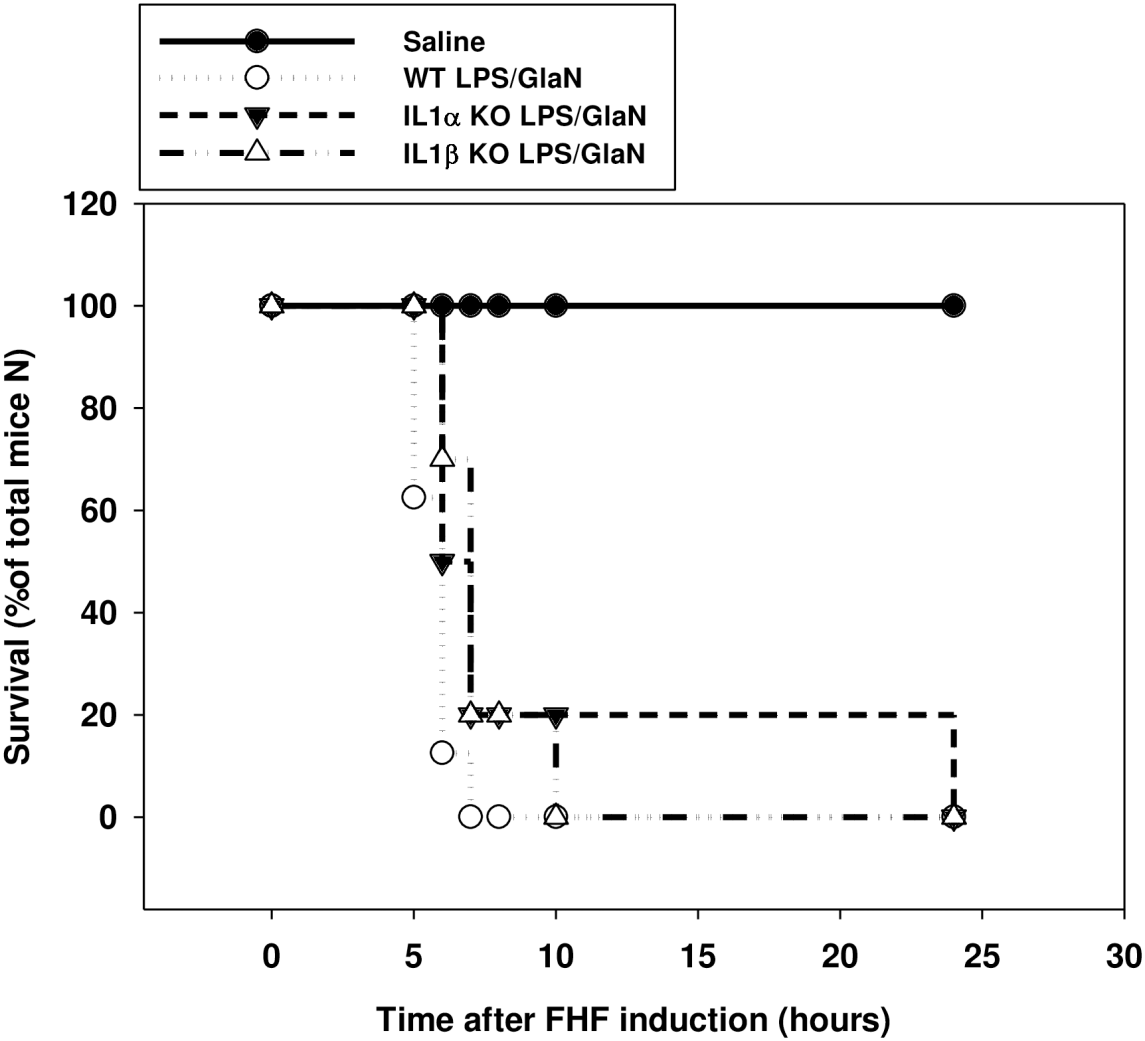
Deficiency of IL-1α or IL-1β prolonged survival of mice following the induction of FHF compared to WT mice. WT IL-1α and IL-1β KO mice were injected with either saline or LPS/GalN at time point 0. Mice were inspected every 30 minutes and the survival rates were calculated as percentage of mice survived from the total number of mice in the group (n = 5 in the saline injected group and n = 8 in the LPS/GalN injected groups).

FHF induction in WT mice was followed by 87.5% (7/8) death within 6 hours and 100% death within 7 hours. In contrast, 50% (5/10) of IL-1α KO mice remained alive for 6 hours after treatment and 20% (2/10) for 7 hours. Two of the IL-1α KO mice (20%) survived 24 hours after FHF induction (Fig 1). Similar survival rates were observed after the induction of FHF in IL-1β KO mice, with 70% (7/10) still alive after 6 hours, and two mice (2/10) after 8 hours, which later died at 10 hours post-treatment (Fig 1).

### The effect of IL-1α and IL-1β deficiency on FHF-associated liver damage

**Fig 2:**
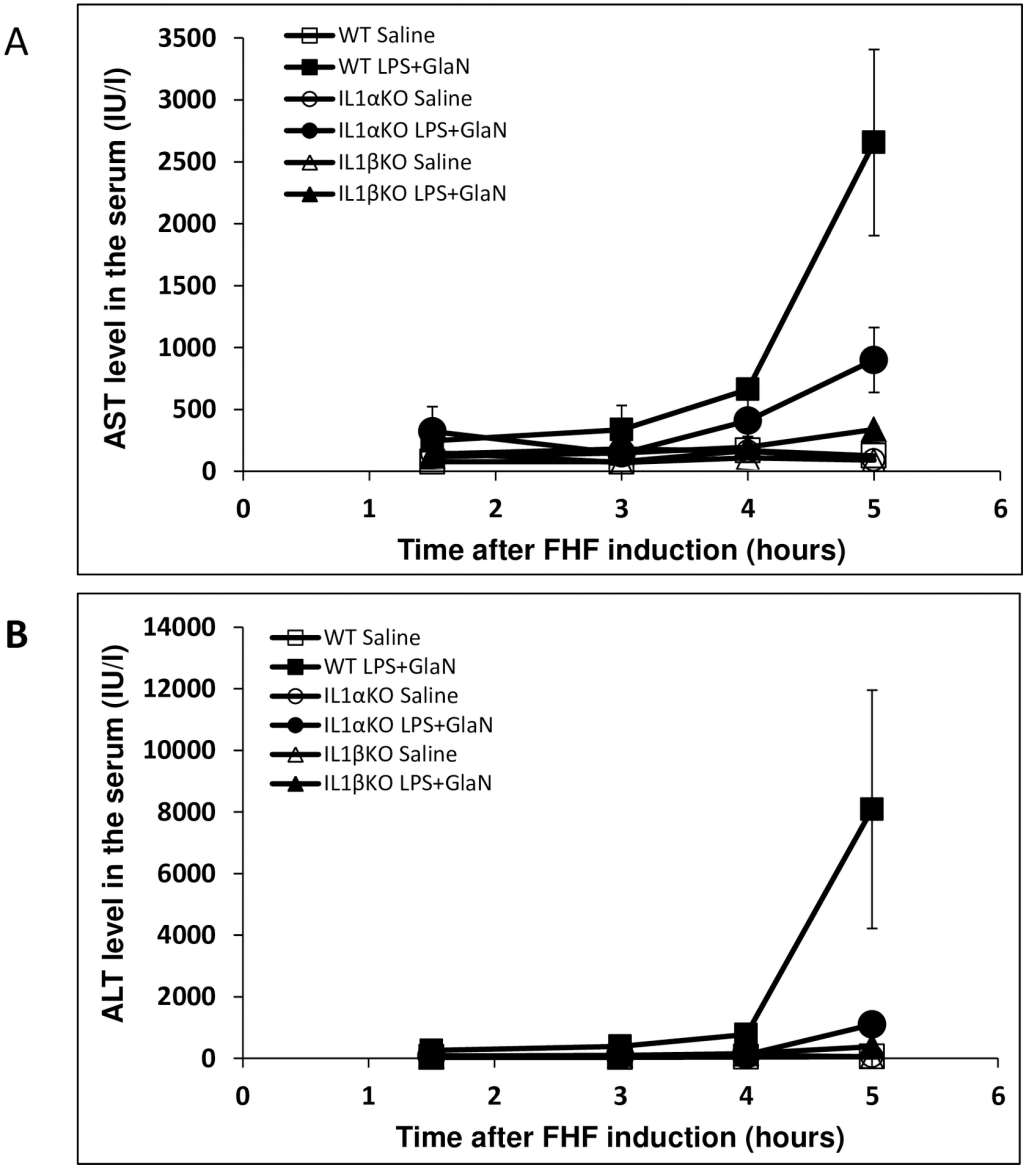
Significant decrease in serum liver enzymes level (AST and ALT) was detected in the IL-1α or IL-1β deficient mice compared to WT mice after FHF induction. WT IL-1α and IL-1β KO mice were injected with either saline or LPS/GalN at time point 0. Blood was collected via the inferior vena cava at the designated times after LPS/GalN injection (n = 3 at each time point of each group). Serum AST (A) and ALT (B) levels were determined using standard procedure. Results are expressed as mean ± standard error. Differences between groups were assessed using the analysis of variance (T-TEST).

**Fig 3:**
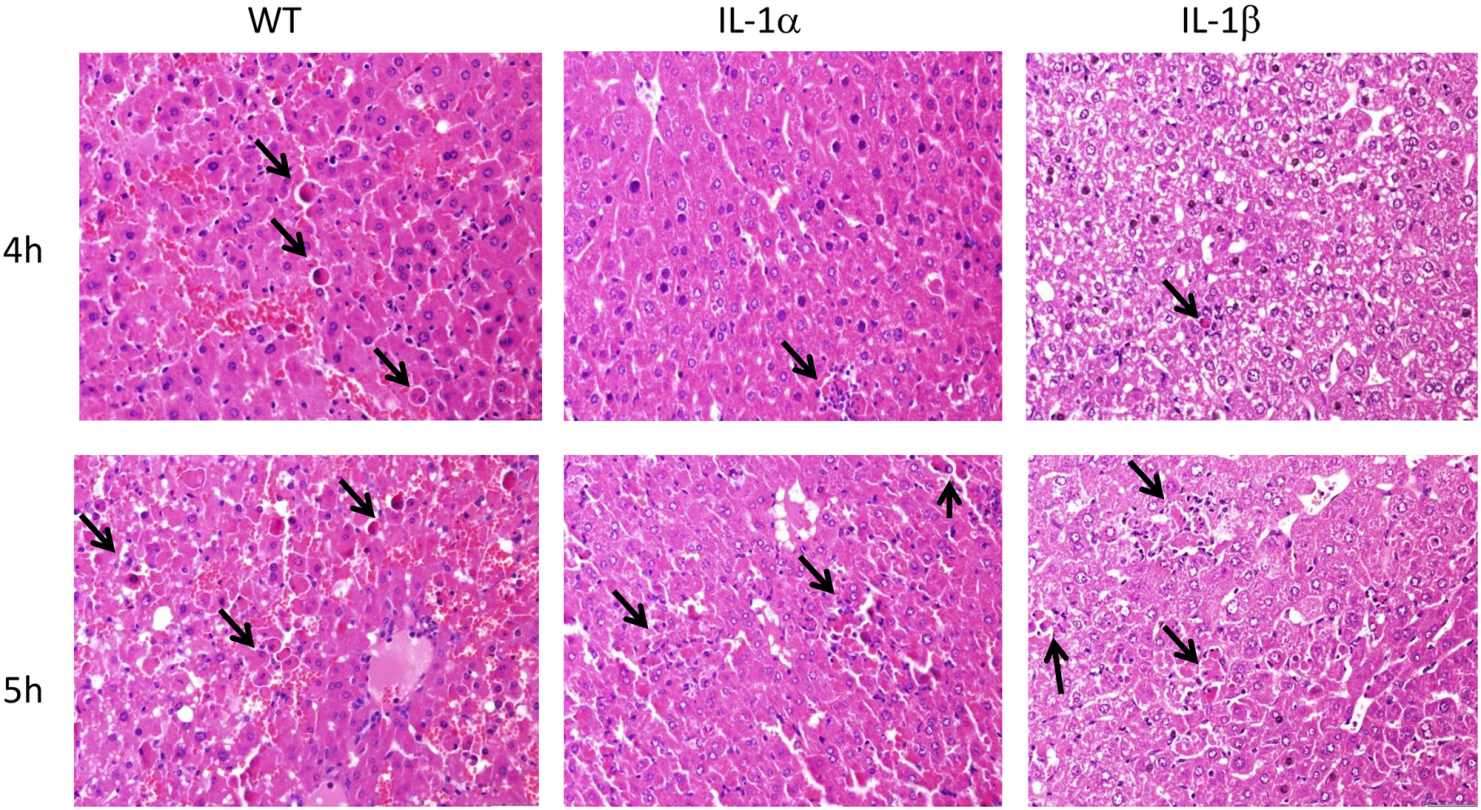
Deficiency of IL-1α or IL-1β delayed FHF-associated liver damage compared to WT mice. Liver specimens were obtained from WT, IL-1α and IL-1β KO mice, 4 and 5 hours after the injection of LPS/GalN. Representative images of H&E-stained sections are shown. Apoptotic bodies (marked with arrows) were observed in livers of WT mice 4 hours after FHF induction (Fig 3A), while less damage and better preserved liver architecture were seen in IL1α in IL-1β KO mice at the same time post-FHF induction (Fig 3C and E). However, 5h following the injections, hepatic damage was also seen in the livers of the IL-1α and Il-1β KO mice.

A slight elevation in serum transaminase, AST and ALT levels was observed within 1.5 hours of FHF induction in WT mice. In contrast, no changes were in the levels of these enzymes were recorded in the IL-1α or IL-1β KO mice. At 5 hours post-induction, serum transaminase levels were significantly higher in treated WT mice reaching mean levels of AST 2,656±752 and ALT 8,083±3,867 IU/L compared to AST 126±22 and ALT 50±4 IU/L in control WT mice (ALT P = 0.0021 and AST P = 0.014317, Fig 2). IL-1α KO mice exhibited slight elevations in enzyme levels 5 hours after FHF induction (AST 899±262 and ALT 1,095±386 IU/L) compared to control-IL-1α KO mice (AST 90±19 and ALT 54±6 IU/L, P = 0.03 and P = 0.05, respectively). At this time point, the reduction in liver enzyme levels was even more pronounced in treated IL-1β KO mice (AST 338±23 and ALT 378±26 IU/L) while in control IL-1β KO mice enzyme levels were AST 90±17 and ALT 48±13 IU/L (P = 0.01 and P = 0.04, respectively). These findings demonstrated attenuated liver damage in IL-1α and IL-1β KO mice in comparison to WT mice. Evaluation of hepatic damage following GalN/LPS administration using H&E staining reinforced the results of the liver enzymes levels. Analysis of the pathology of the livers sections revealed few apoptotic bodies in the hepatic parenchyma in livers of WT mice (Fig 3A), while less damage was seen in IL1α in IL-1β KO mice 4 hours after FHF induction (Fig 3C and 3E). However 5 hours after the LPS/D-Gal injections, apoptotic bodies were observed in discrete aggregates in WT mice, accompanied by few neutrophils. Similar histological findings were also noted in the livers of the IL-1α and IL-1β KO mice.

### IL-1α and IL-1β expression levels in FHF

**Fig 4:**
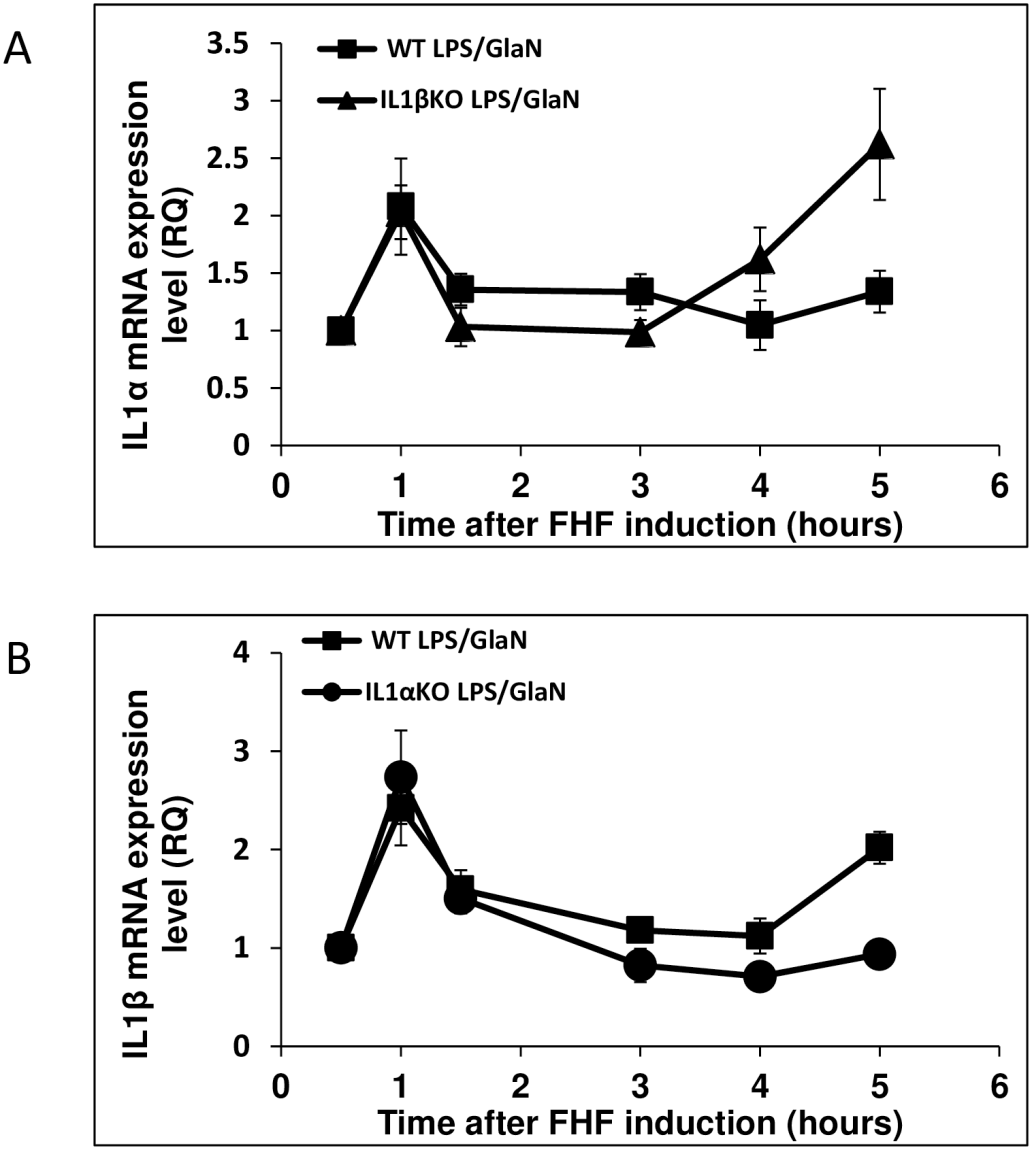
Alterations in hepatic IL-1α and IL-1β expression in IL-1α and IL-1β KO compare to WT mice after LPS/GalN injection. WT, IL-1α and IL-1β KO mice were injected with LPS/GalN at time point 0. Mice were sacrificed at the indicated time points and RNA was purified from their livers. Hepatic IL-1α and IL-1β expression levels were evaluated using qRT-PCR (n = 3 in each group at each time point). Results are expressed as mean ± standard error. Differences between groups were assessed using the analysis of variance (T-TEST).

To better understand the role of IL-1α and IL-1β in the development of FHF, the temporal pattern of the hepatic expression of these cytokines was monitored. Hepatic expression of both IL-1α and IL-1β was up-regulated in WT mice 1 hour after LPS/GalN injection (2.1 and 2.4 fold, respectively, Fig 4). Similar patterns were observed in the KO mice (IL1β in IL1α KO mice (2.7 fold) and IL-1α in IL-1β KO mice (2 fold)) (Fig 4). These transcript levels declined 1.5h post-treatment in all mice groups. However, 5 hours after FHF induction, hepatic expression levels of IL-1α rose again in IL-1β KO (2.6 fold) mice but not in WT mice. At this same time point, an upregulation of IL-1β was observed in WT mice (2 fold), but not in IL-1α KO mice.

### Effect of IL-1α and IL-1β deficiency on the pro-inflammatory pathways induced by LPS/GlaN administration

**Fig 5:**
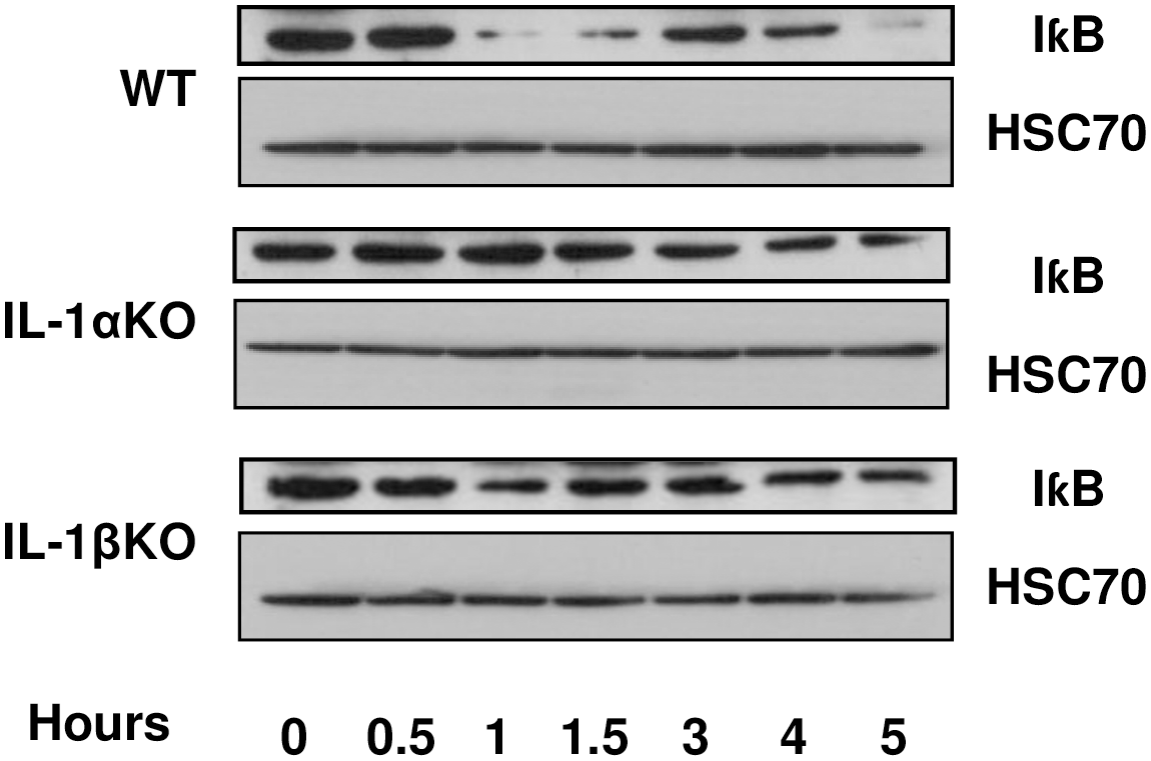
IκB protein levels in the livers of WT IL-1α and IL-1β KO mice after LPS/GalN injection. WT IL-1α and IL-1β KO mice were injected with LPS/GalN at time point 0. Mice were sacrificed at the indicated time points and proteins were purified from their livers. IκB protein levels were analyzed using western blot analysis.

**Fig 6:**
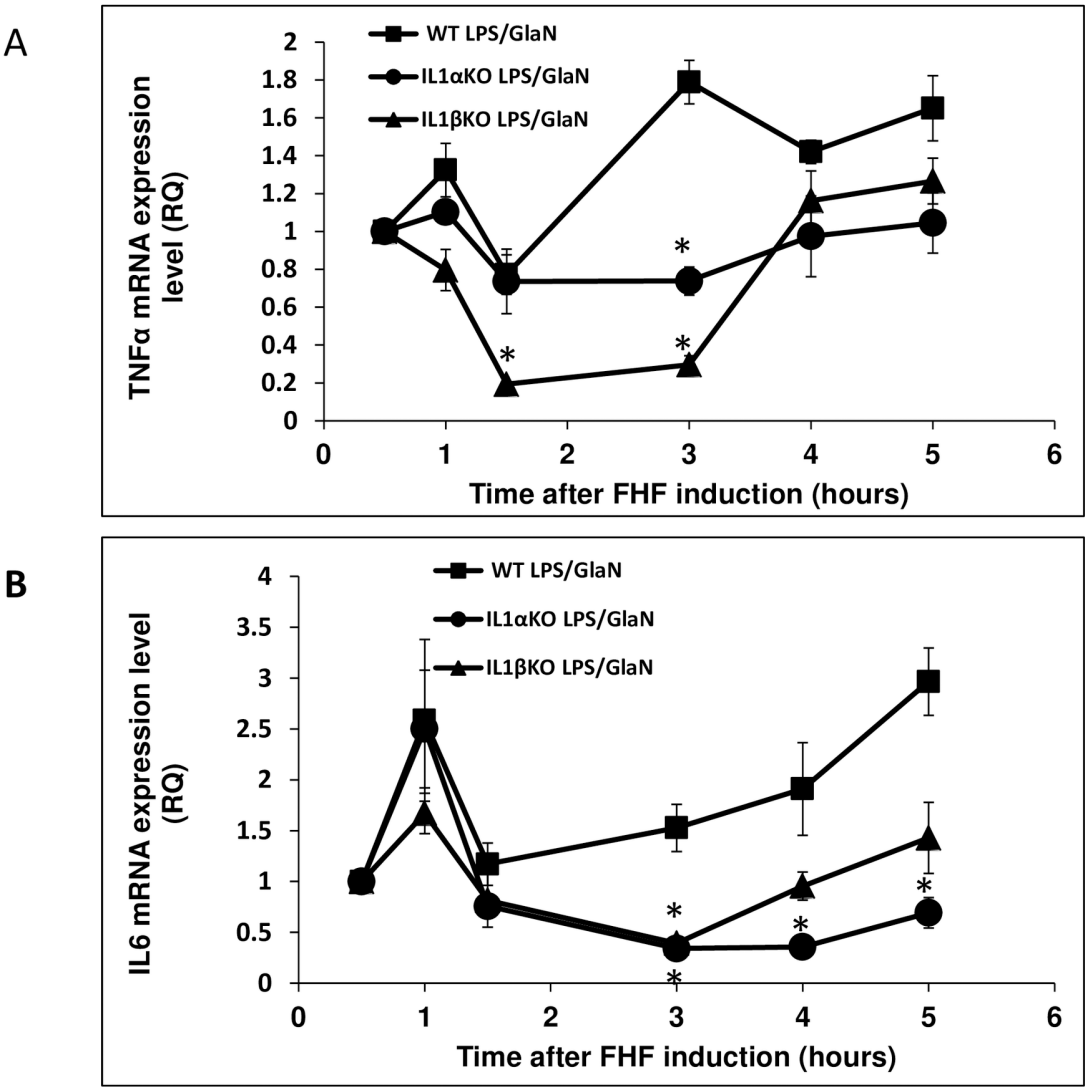
Hepatic TNF-α and IL-6 expression in WT, IL-1α and IL-1β mice levels after LPS/GalN injection. WT, IL-1α and IL-1β KO mice were injected with LPS/GalN at time point 0. Mice were sacrificed at the indicated time points and RNA was purified from their livers. Hepatic TNF-α (A) and IL-6 (B) expression levels were evaluated using qRT-PCR (n = 3 in each group at each time point). Results are expressed as mean ± standard error. Differences between groups were assessed using the analysis of variance (T-TEST). Significant differences between the analyzed groups (IL-1α and IL-1β) and the control group (WT) are marked by * and represent Pv<0.05.

In order to assess the involvement of IL-1α and IL-1β in the regulation of the NFκB pathway during FHF induction, IκB protein levels in mouse livers were determined. IκB levels were very low 1.5 hours post-FHF induction in WT mice, suggesting an active NFκB signaling pathway in these livers (Fig 5). In contrast, IκB levels remained unchanged in treated-IL-1α KO mice and were slightly decreased in IL-1β KO mice. Since the NFκB transcription factors play a critical role in triggering a wide range of pro-inflammatory genes, we then set out to measure the expression levels of IL-6 and TNFα in treated mice. Both TNFα and IL-6 were upregulated one hour after LPS/GlaN administration to WT and IL-1α KO mice. However, no elevation in TNFα levels was observed in IL-1β KO mice, and only a slight increase in IL-6 expression was detected (Fig 6). Within 1.5 hours of LPS/GlaN injection, hepatic expression levels of these genes returned to normal in WT and IL-1α KO mice. However, in WT mice, these genes were upregulated again 3–4 hour later and reached their highest levels 5 hours after FHF induction. As previously mentioned, all WT mice died within 5–6 hours, coinciding with the peak expression levels of these pro-inflammatory genes. The transcript levels of these genes declined also in livers of IL-1 KO mice, after an initial elevation at 1 hour post-treatment. However, the second transactivation of these genes was delayed by one hour in IL-1 KO mice and their expression levels were significantly lower compared to WT mice.

### The effect of IL1α and IL1β deficiency on apoptosis progression following FHF induction

**Fig 7:**
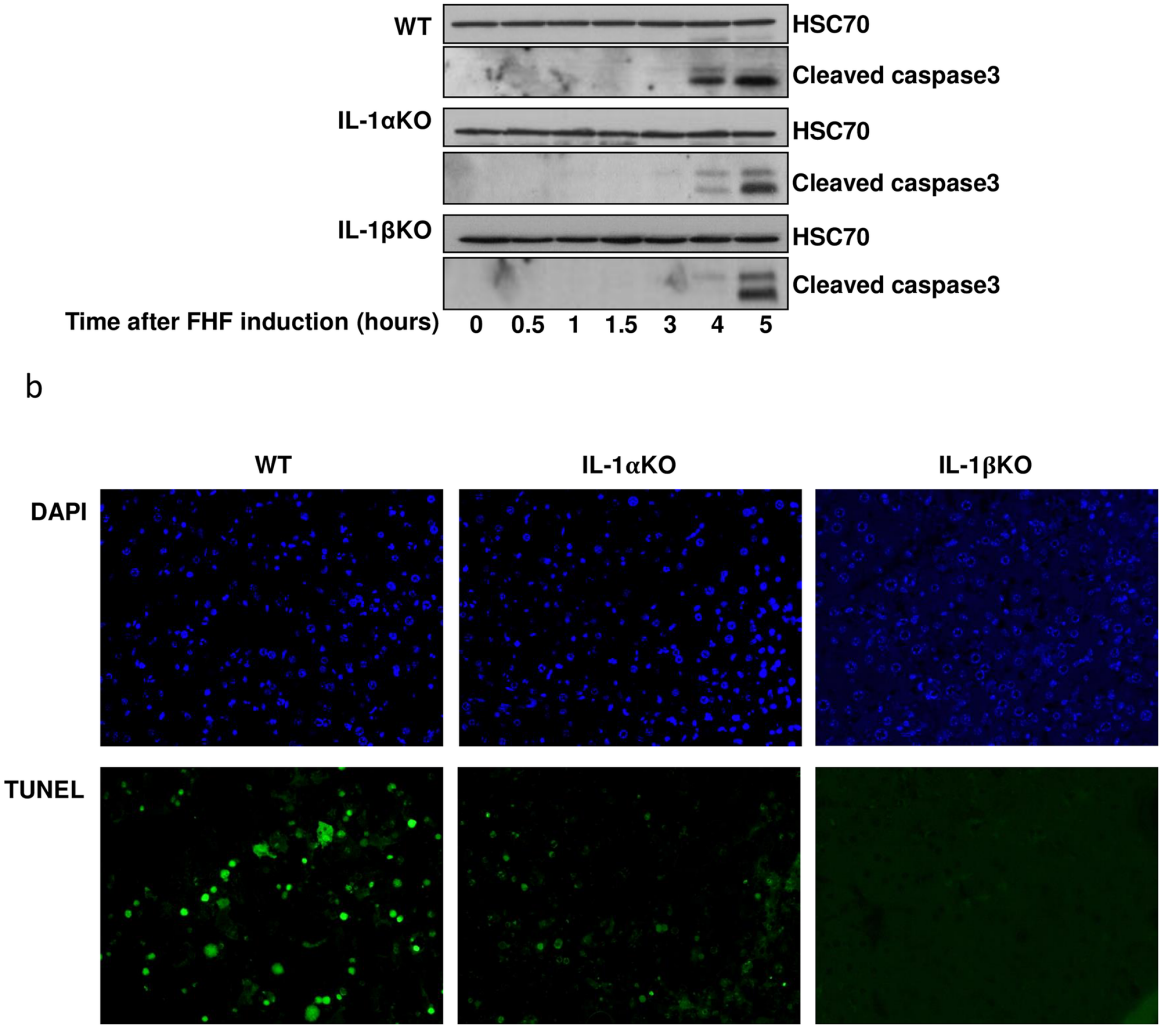
Significant decrease in apoptosis was detected in the IL-1α or IL-1β deficient mice compared to WT mice after FHF induction. (A) WT IL-1α and IL-1β KO mice were injected with LPS/GalN at time point 0. Mice were sacrificed at the indicated time points and proteins were purified from their livers. The hepatic expression of the active, cleaved, Caspase 3 was detected using western blot analysis. (B) WT, IL-1α and IL-1β KO mice livers were harvested 5 hours after LPS/GalN injection and imbedded in paraffin blocks. Apoptotic cells were identified using the DeadEndTM flourometric TUNEL system.

To evaluate the effect of IL-1α and IL-1β on apoptosis in our FHF model, cleaved caspase 3 and DNA fragmentation levels were monitored. While liver caspase 3 cleavage was detected 4 hours after LPS/ GalN treatment in WT mice, its activation was detected only 5 hours after treatment in the IL-1α and IL-1β KO mice (Fig 7a). Accordingly, DNA fragmentation was high in the livers of WT mice 5 hours after treatment with LPS/GalN, but not in the livers of IL-1α and IL-1β KO mice (Fig 7b). In the IL-1β KO mice we could not detect any DNA fragmentation 5 hours after FHF induction (Fig 7b).

## Discussion

To the best of our knowledge, the current study is the first to describe the time-dependent alterations in the expression of hepatic pro-inflammatory cytokines and apoptotic markers in the context of LPS/GalN-induced FHF. Additionally, the study focused on the independent role of IL-1α and IL-1β in the pathogenesis of FHF. Our results demonstrated a similar expression pattern for all the tested pro-inflammatory cytokines after the induction of FHF in WT mice. An initial acute increase in expression levels was detected one hour post-treatment, and seemingly corresponded to a direct response to the LPS injection, as previously demonstrated both in vitro and in vivo [32, 33]. At 1.5 hours post-treatment, the expression levels of all the pro-inflammatory cytokines dropped to baseline. Similar findings were reported by Furuya et al., who induced FHF, with a much milder protocol (2.5 μg/kg LPS and 300 mg/kg GalN), in mice, and detected an upregulation in circulating serum IL-6 and TNFα 1 hour after treatment, which then dropped within 1-2h of treatment [34]. In our model, the expression levels of pro-inflammatory cytokines rose again 3–4 hours after FHF induction, which coincided with their death. Furuya et al. did not report such an elevation at these time points, likely due to their less aggressive model, in which only 20% of the mice died 8 hours post-treatment [34]. In pursuit of the molecular mechanism underlying the second elevation in pro-inflammatory gene expression, we examined the NFκB pathway, a signaling pathway that plays a key role in the activation of the pro-inflammatory mechanism in FHF [35, 36]. NFκB activity is tightly regulated by its inhibitor IκB. In quiescent cells, IκB binds NFκB, consequently preventing its translocation to the nucleus and binding to the DNA. Upon stimulation, IκB is phosphorylated and degraded; thereby releasing NFκB to enter the nucleus, bind to the DNA and promote transcription of pro-inflammatory genes. In our FHF model, the levels of IκB in WT mice decreased 1–1.5h after FHF induction, and paralleled the upregulation of NFκB signaling pathway. These events preceded the second rise in the pro-inflammatory cytokine expression, which led us to suggest that NFκB activation regulates the re-elevation of the pro-inflammatory cytokines 3–4 hours after the induction of FHF and until death.

Upon liver injury, both survival and death signals are co-activated in each hepatocyte and the cell fate is determined by the balance between these pathways [37]. Apoptosis in the livers of FHF WT mice was activated only 3 hours after FHF induction. One of the central signaling routes leading to cellular apoptosis in hepatocytes is mediated by TNFα [38–40]. Indeed, an acute increase in TNFα levels was observed in WT mice, 2–3 hours after FHF induction, followed by caspase 3 processing and eventually DNA fragmentation.

Since the pro-inflammatory process plays a significant role in the development of FHF, we chose to study the independent roles of IL-1α and IL-1β in the process. It has already been shown that inhibition of the IL-1 pathway can improve survival and lower the expression levels of liver enzymes after FHF induction [29]; Another work has demonstrated a correlation between IL1 and MMP9 expression, upon the induction of FHF, that was implicated in ECM degradation, sinusoidal collapse, leading to parenchymal cell death, and loss of liver function [41] however, little is known about the specific roles of IL-1α and IL-1β in the process. Our results demonstrated that both IL-1α and IL-1β play an important role in the development and acceleration of FHF-associated inflammation and apoptosis. IL-1α and IL-1β KO mice demonstrated extended survival and less liver injury after FHF induction compared to FHF WT mice. Furthermore, we have shown that deletion of either IL-1α or IL-1β delayed the upregulation of hepatic IL-6 and TNFα following LPS/GalN administration. In WT mice, IL-1α and IL-1β upregulated the pro-inflammatory process through a dramatic decrease in hepatic IκB levels. In contrast, only a slight decrease in IκB levels was detected 1.5 hours after FHF induction in IL-1β KO mice, while no change was recorded in the IL-1α KO mice. It is unlikely that this slight reduction in the IL-1β KO mice was responsible for the resurge in expression of the pro-inflammatory cytokines 4–5 hours after FHF induction; other signaling pathways might be involved in this phenomenon.

Apoptosis is a prominent feature of liver diseases and it has been shown that the regulation of hepatic apoptosis affects the progression of liver diseases [42]. Investigating this process in our model we have noticed that apoptosis developed earlier and to a higher extent in the WT mice compared to the IL-1α and IL-1β KO mice. As previously mention this result is probably a consequence of the delay in the expression of TNFα and MMP9 (S1 Fig) in the IL1α and IL1β KO mice [41].

Although previous published studies showed mutual induction of IL-1α and IL-1β [23, 30], our results indicate that LPS/GalN-induced FHF results in a rise in IL-1α expression in IL-1β KO mice and IL-1β expression in IL-1α KO mice was similar to the levels measured within 1 h of FHF induction in WT mice. These observations indicate that in this initial stage of FHF stimulation, there is no mutual induction between the two genes. Additionally, IL-1α levels in IL-1β KO mice 5 hours following FHF induction were even higher than in the WT mice. However, in IL-1α KO mice, a reduction in IL-1β mRNA levels was observed 3–5 hours after FHF induction. These results suggest that in our FHF model, IL-1α expression regulation is independent of IL-1β and it might even be transcribed to a higher level to compensate for the absence of IL-1β. In contrast, IL-1β transcription is dependent on IL-1α expression.

In conclusion, this study illustrated the contribution of both IL-1α and Il-1β in the development and acceleration of fulminant hepatitis failure. However, despite the reduced inflammation, hepatic injury and delayed lethality recorded in IL-1 KO mice treated with LPS/GalN, neither gene deletion rescued the phenotype. These data suggest that IL-1α or IL-1β might have a similar and likely complementary role in FHF. Both are associated with the activation of the NFκB pathway, upregulation of pro-inflammatory mechanisms, and induction of apoptosis, liver damage and death. Thus, while each of these cytokines may serve as therapeutic potential target for FHF, additional studies evaluating the role of both interleukins in FHF models are still necessary. Furthermore, in this study we have determined the expression pattern of pro-inflammatory cytokines during FHF progression. We believe that these new insights into FHF progression may pave the road to the development of new targets for inhibition of this disease.

## Supporting information

S1 Fig Alterations in hepatic MMP9 expression in IL-1α and IL-1β KO compare to WT mice after LPS/GalN injection. WT, IL-1α and IL-1β KO mice were injected with LPS/GalN at time point 0. Mice were sacrificed at the indicated time points and RNA was purified from their livers. Hepatic MMP9 expression levels were evaluated using qRT-PCR (n = 3 in each group at each time point). Results are expressed as mean ± standard error.
(TIF)
