# Supplementary figures and images for: Interleukin-1α and Interleukin-1β play a central role in the pathogenesis of fulminant hepatic failure in mice
Source: PLoS One. 2017 Sep 27;12(9):e0184084. doi: 10.1371/journal.pone.0184084 (PMC5617151; doi:10.1371/journal.pone.0184084)

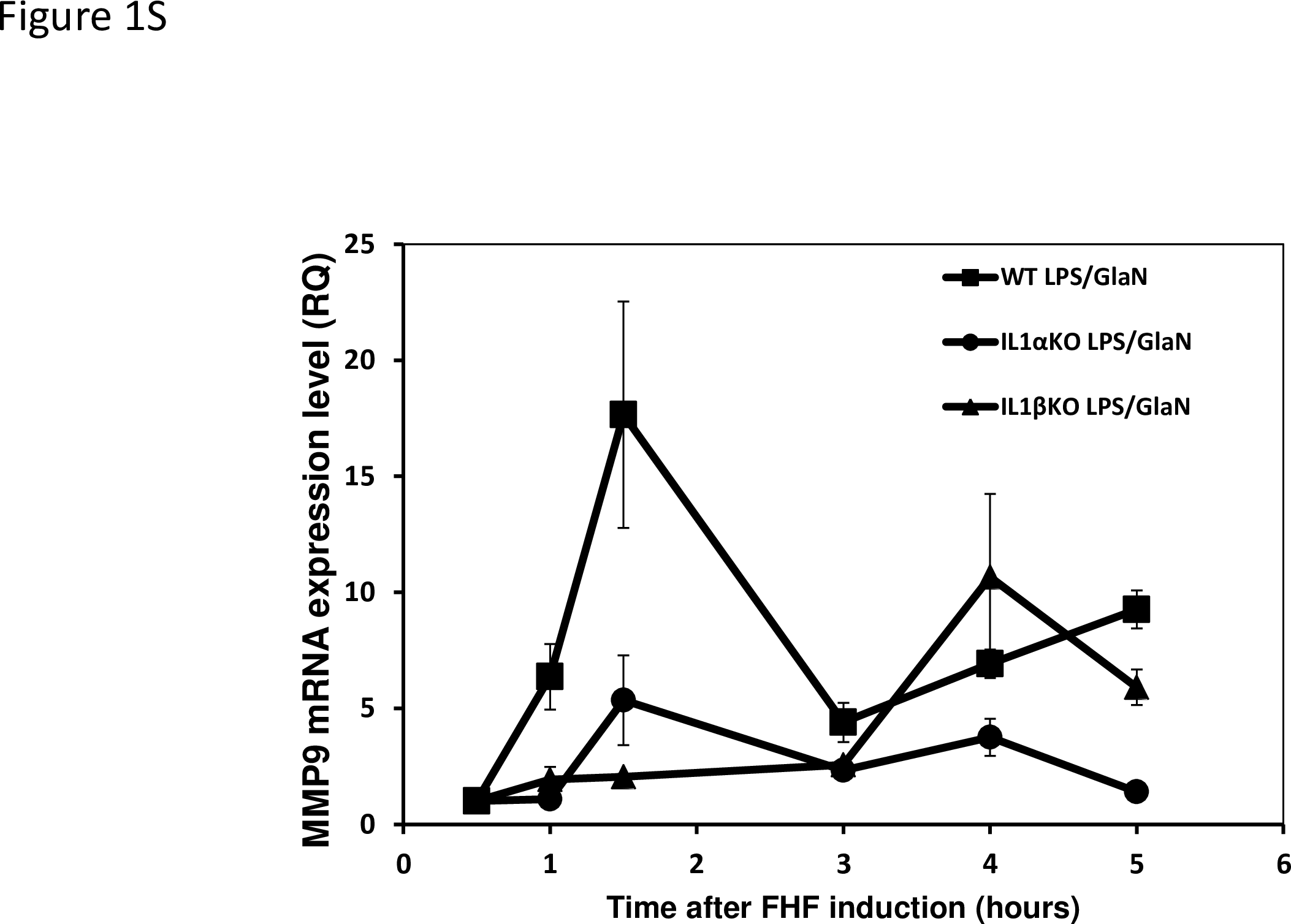

Supplement: S1 Fig — WT, IL-1α and IL-1β KO mice were injected with LPS/GalN at time point 0. Mice were sacrificed at the indicated time points and RNA was purified from their livers. Hepatic MMP9 expression levels were evaluated using qRT-PCR (n = 3 in each group at each time point). Results are expressed as mean ± standard error. (TIF) [file pone.0184084.s001.tif]
